# The Susceptibility of Coccydynia in Morphology: A Cross-Sectional Study

**DOI:** 10.1155/prm/2932223

**Published:** 2025-10-13

**Authors:** Hang Feng, Xin Chen, Chen Cao, Dongqin Wan, Hongming Liu, Eryang Zhang, Zhenghong Yu

**Affiliations:** ^1^Department of Surgery of Spine and Spinal Cord, Henan Provincial People's Hospital, Zhengzhou, China; ^2^Department of Orthopedic, Yuncheng Central Hospital, Yuncheng, Shaanxi, China; ^3^Department of Radiology, Henan Provincial People's Hospital, Zhengzhou, Henan, China

**Keywords:** coccydynia, coccyx, morphology, morphometry, radiography, sacrum

## Abstract

**Background:**

Coccyx morphologic features have been found to be associated with coccydynia, while coccygeal anatomical data of patients with coccydynia is scarce. The purpose of this study is to evaluate the morphological and morphometric parameters of the sacrococcyx in patients experiencing coccydynia.

**Methods:**

Radiographic examinations of the pelvis were conducted on a cohort of 244 patients diagnosed with coccydynia. Sacrococcygeal morphological features and morphometric parameters were documented and measured.

**Results:**

The most prevalent coccyx type was Type II (33.1%), followed by Type III (28.1%), Type IV (21.5%), Type I (12%), and Type V (5.4%), respectively. Coccygeal sacralization was present approximately one-third of cases. Around a quarter of the subjects exhibited subluxation. Bony spicule was present in 22.3% individuals. Lateral deviation of the coccyx was present in roughly one-fifth of the patients. Sacral curvature index and sacrococcygeal angles were greater in males with coccydynia than in females, while the coccygeal curvature index was smaller in males. The straight length of the coccyx, coccygeal curved index, and sacrolumbar angle all showed an increase with age.

**Conclusion:**

Patients with coccydynia usually showed a more ventrally curved coccyx. Bony spicule, coccygeal sacralization, and lateral deviation of the coccyx were common among coccydynia patients. Coccyx alignment tended to become straighter, and the sacrolumbar angle tended to increase with age. Individuals presenting with these aforementioned anatomical features appear to be predisposed to coccydynia.

## 1. Introduction

Coccydynia, characterized by pain in or around the coccyx, is typically exacerbated by activities such as sitting, standing, and walking [1, 2]. The primary cause of coccygodynia often stems from direct or indirect trauma to the coccyx or its adjacent structures, potentially leading to instability in the sacrococcygeal or intercoccygeal joints [3]. Other contributing factors encompass a range of etiologies, including tumors, infections, disc degeneration, and coccygeal nerve entrapment, among others [4, 5]. In approximately one-third of cases, coccydynia is classified as idiopathic, meaning it is not associated with any well-defined pathological condition [6]. Furthermore, both obesity and female sex are recognized as predisposing factors for coccydynia [3].

Many researchers have paid attention to potential abnormalities in the bony anatomy of the coccyx as a cause of coccydynia. Previous studies have reported that individuals are predisposed to coccygodynia when there is evidence of subluxation in the sacrococcygeal or intercoccygeal joints [7]. Additionally, Woon et al. [8] found that patients with a pronounced ventral angulation of the coccyx are more likely to develop coccydynia, while Ahmed Shamset et al. [9] suggested that the presence of bony spicules on the coccyx increases susceptibility to coccydynia. However, it is important to note that coccygeal anatomical data for patients with coccydynia are currently limited, and research examining the relationship between the morphology and morphometry of the coccyx and coccydynia remains insufficient.

Hence, the primary objective of the current study was to comprehensively elucidate the morphological and morphometric features of the coccyx in individuals suffering from coccydynia. Furthermore, we aimed to investigate whether there are correlations between gender and age and measurements in the group examined.

## 2. Materials and Methods

### 2.1. Subjects and Setting

We conducted a retrospective cross-sectional study involving patients who sought medical care for sacrococcygeal pain during the period from January 2021 to December 2022. Individuals with a history of sacral and/or coccyx fractures, tumors, infections, or congenital disorders were excluded from the study. The research protocol underwent thorough review and received approval from the institutional ethical committee of the authors.

All patients underwent pelvis anteroposterior and lateral X-ray examinations while in the supine position. Scans were obtained with the Siemens Ysio Max X-ray Digital Radiography (DR) Systems (Siemens Healthcare, Erlangen, Germany). A series of exposure sequences were captured by adjusting the tube voltage (kV) within the range of 60–120 kV and the tube load (mAs) value within the range of 0.5–25.0, respectively.

**Figure 1:**
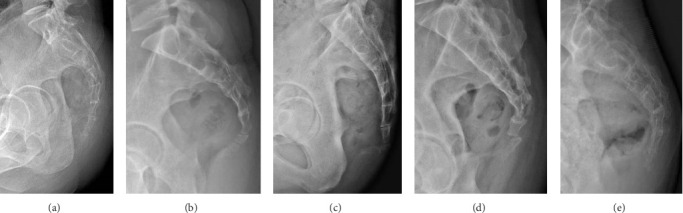
Types of coccyx: (a) Type I, (b) Type II, (c) Type III, (d) Type IV, and (e) Type V.

**Figure 2:**
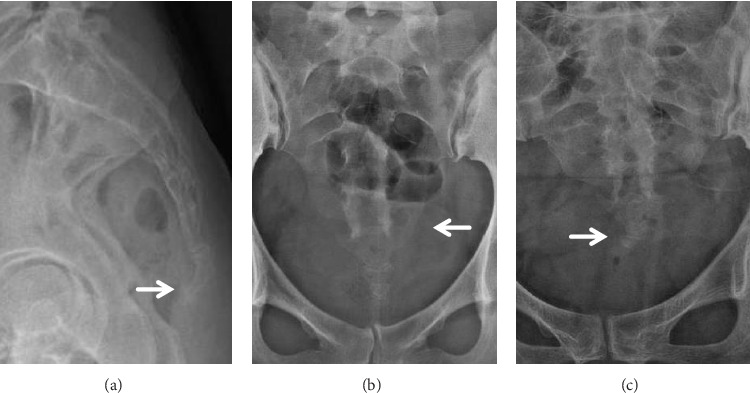
Examples of coccygeal morphologic findings. (a) Bony spicule. (b) Unilateral coccygeal sacralization. (c) Lateral deviation of the tip of the coccyx.

**Figure 3:**
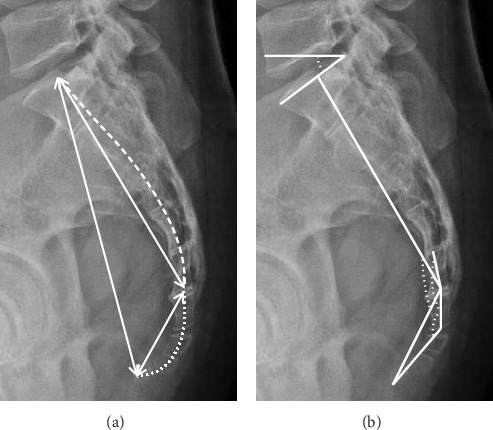
Schematic diagram of morphometry in the sacrococcygeal region in sagittal view. (a) Straight (solid line) and curved (dotted line) lengths of sacrum and coccyx. (b) Angle measurements of the coccyx and sacrum: sacrococcygeal angle, sacrococcygeal joint angle, intercoccygeal angle, and sacrolumbar angle.

We conducted an analysis of both qualitative and quantitative parameters pertaining to sacrococcygeal anatomy, and the definitions for these parameters were outlined in Table 1 and illustrated in Figures 1, 2, and 3. The classification of coccyx type was based on the modified Postacchini and Massobrio classification [5, 7]. All measurements were acquired by two experienced radiologists and orthopedists. To assess both intraobserver and interobserver reliability, 16 randomly selected patients underwent measurements on two separate occasions with a two-week interval, and the reliability was evaluated using the intraclass correlation coefficient (ICC). In accordance with stages of growth and development, child-bearing age, and the period of menopause, the present study categorized individuals under 21 years old as the adolescent group, those over 50 years old as the elderly group, and those falling between these age brackets as the middle-young-aged group. We then analyzed the differences in these parameters with respect to age and gender matching.

### 2.2. Statistical Analysis

Statistical analyses were performed using the SPSS software (version 22.0, SPSS Inc., Chicago, IL, USA). Continuous variables were presented as mean ± standard deviation, while categorical variables were expressed as frequency distributions and percentages. To assess differences between two groups with normally distributed data, *T*-tests were employed. For multiple comparisons, ANOVA was utilized. In cases where the data exhibited heterogeneity of variance, nonparametric tests were applied. Associations among qualitative variables were explored using *χ*^2^ and Fisher's exact test. The threshold for statistical significance was set at *p* < 0.05. Graphs were generated using GraphPad Prism 5 (GraphPad Software, Inc., San Diego, CA, USA).

## 3. Results

**Figure 4:**
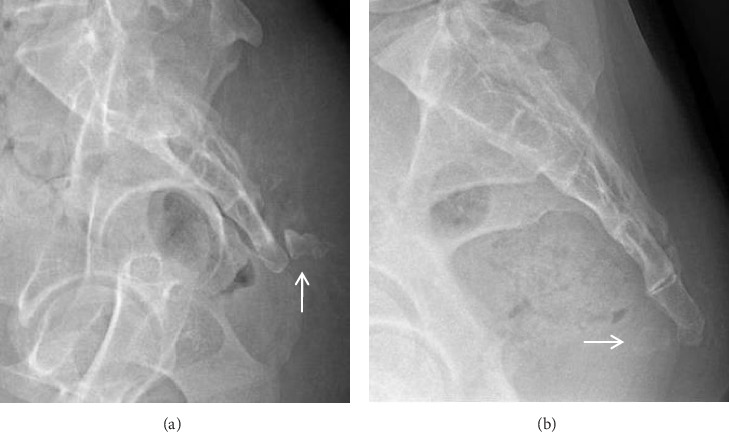
(a) Complete posterior dislocation of the coccyx. (b) Complete anterior dislocation of the coccyx.

The study encompassed a cohort of 244 participants, comprising 80 (32.8%) males and 164 (67.2%) females. The mean age across the entire cohort was 35.42 ± 15.18 years, with males averaging 33.23 ± 15.36 years and females averaging 36.5 ± 15.02 years. The distribution of participants across age groups, categorized by decades of life, included 36 individuals in the adolescent group, 168 in the middle-young-aged group, and 38 in the elderly group. Notably, due to the presence of complete sacrococcygeal dislocation in two female patients (aged 33 and 35 years, respectively; Figure 4), a total of 242 patients were included in the subsequent analysis of coccyx morphology and morphometry.

### 3.1. Reliability

The ICCs for both intra- and interobserver reliability consistently exceeded 0.75 for all quantitative measurements. This signifies a high level of reproducibility and repeatability in the study's measurements.

### 3.2. Coccygeal Morphology

In this study, the composition of the coccyx varied, comprising either two (8.3%), three (44.6%), four (38.3%), or five (8.3%) vertebrae. No statistically significant differences were observed in terms of gender or age in relation to the number of vertebrae (*p* > 0.05). Regarding the classification of coccyx types among the patients, type II was the most common (33.1%), followed by type III (28.1%). Types I, IV, and V accounted for 12%, 21.5%, and 5.4%, respectively. There were no significant differences in coccygeal type based on gender or among the age groups (*p* > 0.05), as detailed in Tables 2 and 3.

Coccygeal sacralization, characterized by fusion between the sacral and transverse processes, was observed in 75 (31%) of the subjects. There were no significant differences noted in terms of unilateral or bilateral fusion. Coccygeal sacralization types did not display any significant variations within different age or gender groups (*p* > 0.05). Joint fusion was prevalent in 57% of the subjects, with the sacrococcygeal joint affected in 15.3% and the intercoccygeal joint in 41.7%. There were no significant associations found between the presence of joint fusion and the subjects' sex or age (*p* > 0.05). On the contrary, sacrococcygeal and intercoccygeal subluxations were relatively infrequent, affecting 8 (3.3%) and 43 (17.7%) subjects, respectively. Notably, the majority of intercoccygeal subluxations primarily involved the first intercoccygeal joint (62.8%) (Tables 2 and 3).

A bony spicule was identified in 54 (22.3%) of the coccyges, and there were no significant gender or age-related biases observed in this regard (Tables 4 and 5). Regarding the lateral deviation of the coccyx, cases with a deviation angle of less than 10° were categorized as the “straight type.” Among the patients, a total of 47 (19.4%) exhibited a laterally deviated coccyx, with a mean coccygeal deviation angle of 16.87 ± 6.35°. While the incidence of laterally deviated coccyx was higher among females (14.5%) compared to males (5%), this disparity did not reach statistical significance (*p* > 0.05). However, it's worth noting that coccygeal deviation was more prevalent among middle-young-aged patients, showing statistical significance (*p* < 0.05) (Tables 2, 3, 4, and 5).

### 3.3. Sacrococcygeal Morphometry

The mean straight lengths of the sacrum, coccyx, and sacrococcygeal segment measured 135.1 ± 13.77, 40.37 ± 7.92, and 156.85 ± 19.29 mm, respectively. Meanwhile, the mean curved lengths of these structures were 143.74 ± 13.92, 46.68 ± 9.16, and 190.42 ± 18.13 mm, respectively. Male patients exhibited significantly longer sacrums and sacrococcygeal segments than their female counterparts (*p* < 0.001). There was no significant correlation between coccygeal length and gender (*p* > 0.05). Furthermore, a significant trend was observed indicating that both coccyx straight and curved lengths increased with age (*p* < 0.05). In contrast, sacral lengths and sacrococcygeal straight lengths did not show significant associations with age, while sacrococcygeal curved lengths exhibited a weak positive correlation (*p*=0.031) (Tables 4 and 5).

Among male patients, both sacral and sacrococcygeal curvature indices were significantly greater than those in female patients, whereas the coccygeal curvature index was significantly lower (*p* < 0.001). Additionally, as age increased, the coccygeal curvature index showed a significant rise (*p*=0.002), indicating that older patients tended to have straighter coccyges. The average sacrococcygeal, sacrococcygeal joint, and intercoccygeal angles measured 113.47° ± 12.76°, 167.96° ± 9.96°, and 129.51° ± 18.52°, respectively. Significant gender differences were observed in the sacrococcygeal and sacrococcygeal joint angles, which were notably larger in males than that in females (*p* < 0.05). Although not statistically significant, the intercoccygeal angle tended to be smaller in males than in females. These findings, consistent with the curvature index, suggest that the coccyx in male patients tends to exhibit a greater ventral curvature, while the sacrum appears straighter in comparison to females. Moreover, the sacrolumbar angle displayed an increase with age, indicating a fair positive correlation (*p*=0.003) (Tables 4 and 5).

## 4. Discussion

In this research, we conducted a comprehensive and meticulous examination of coccygeal morphology and morphometry in patients with coccydynia. Previous studies have reported a 4 to 5 times higher incidence of coccydynia in females than in males [6], whereas our findings indicate that it is approximately two times more prevalent in females. The higher incidence in women may be attributed to distinct anatomical characteristics in females, coupled with the impact of childbirth [3, 10].

Among our subjects, the most frequent number of coccygeal segments observed was three, followed by the presence of four. Previous research has suggested that the number of coccyx segments may exhibit ethnic differences, potentially attributed to intercoccygeal or sacrococcygeal fusion [11]. In our study, we observed that fusion of the sacrococcygeal and intercoccygeal joints was common, present in more than half of our subjects, which aligns with the findings from other studies [12–14]. The high fusion prevalence presents a paradox, as fusion typically implies stability. We propose this warrants investigation into segment-specific fusion patterns and their biomechanical impact on adjacent mobile segments. The literature on the relationship between coccydynia and coccygeal segments or joint fusion is inconclusive. Some authors suggested that reduced intercoccygeal joint fusion may predispose individuals to coccydynia [8], while others found that patients experiencing coccydynia had a similar number of coccygeal segments compared to normal individuals [7].

The coccygeal sacralization was common and present in about one-third of our subjects. The fifth sacral nerve ventral rami pass over the first coccygeal transverse processes, and the ventral rami of the coccygeal nerve pass anterolaterally beneath the first coccygeal transverse processes [15]. Given the close relationship between the coccygeal transverse processes and the ventral rami of the sacral and coccygeal nerves, this fusion could potentially contribute to nerve irritation or entrapment, a hypothesis worthy of future investigation.

In this study, Type II coccyx was the most prevalent among patients with coccydynia, followed by Type III, while Type I was significantly less common. Given that Type II and III coccyxes exhibit more pronounced forward-pointing angulation compared to Type I, our findings suggest that patients experiencing coccydynia tend to have a more ventrally curved coccyx. These results are generally consistent with the findings previously reported in literature studies. Ahmed Shams et al. [9] compared the coccygeal features of patients with and without coccydynia using magnetic resonance imaging (MRI) and reported Type II as the most common morphology in their coccydynia cohort. Another MRI study reported patients with coccydynia typically had a more ventrally curved coccyx compared to individuals without coccydynia [8]. Woon et al. [8], using MRI, similarly found a higher prevalence of more ventrally curved types in symptomatic patients compared to controls, supporting the notion that ventral curvature is associated with coccydynia. The foundational X-ray work by Postacchini and Massobrio similarly identified a higher prevalence of curved Types (III-VI) in patients undergoing coccygectomy compared to asymptomatic individuals [7]. The low prevalence of the straighter Type I coccyx (12%) in our symptomatic cohort further supports the notion that ventral angulation predisposes individuals to pain. Besides, the presence of a retroverted Type V coccyx (5.4%), though less frequent, represents a distinct morphological variant that can cause pain through direct pressure on subcutaneous tissues [16].

The morphometric findings suggest that males with coccydynia tend to have longer and straighter sacra and sacrococcyx, while their coccyges are more ventrally curved. These findings above were consistent with those in several prior studies, but contrary to those in Arab subjects without coccydynia [8, 12, 13, 17]. The coccygeal curvature index significantly increased with age in our study, indicating a tendency toward a straighter coccyx in older patients. This finding resonates with CT-based studies: Yoon et al. [11] observed a similar age-related straightening trend in a Korean population, and Marwan et al. [12] reported a higher proportion of straight coccyges in older Arab adults. This age-related straightening may reflect degenerative changes or altered biomechanics in older adults. While direct comparison of curvature index values between our X-ray study and these CT studies is cautioned against due to differences in measurement technique and patient positioning, the consistency in the observed trend across modalities and populations strengthens the evidence for age-related morphological changes in the coccyx. Although both women and men can suffer from coccydynia, it occurs more commonly in women. This relationship may be attributed to the fact that coccydynia is more common in women, with vaginal delivery being a significant contributing factor to coccydynia [3]. Moreover, it has been demonstrated that women with a history of vaginal deliveries tend to have a longer coccygeal migration length [18].

Approximately a quarter of the subjects in our study exhibited subluxation in at least one joint, with the first intercoccygeal joints being the most commonly affected. Previous studies revealed that Type IV coccyx and joint subluxation were significantly more prevalent in patients with coccydynia compared to asymptomatic individuals [3, 9, 12, 19]. The presence of joint subluxation can lead to coccygeal hypermobility or dynamic instability, which is considered one of the most common causes of coccydynia.

In our study, Type V coccyx, characterized by a retroverted tip, was present in approximately 5% of patients with coccydynia. Type V coccyx can lead to abnormal weight-bearing forces on the coccyx in the seated position, as it fails to exhibit the slight forward flexion. Instead, it extends somewhat backward, exerting pressure and friction on subcutaneous tissues, which can initiate chronic inflammation in these tissues [16]. Lateral deviation of the coccyx's tip was present in about a fifth of patients in our study. Lateral deviation of the coccyx may be caused by traumatic accidents, such as falls on the buttocks or prolonged sitting in an incorrect or uncomfortable position. The observed lateral deviation of the coccyx may lead to asymmetric forces and pelvic floor dysfunction, suggesting another mechanistic pathway for pain [20].

**Figure 5:**
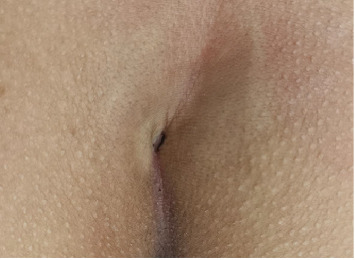
Pit in the skin overlying a spicule.

Our study also revealed that a bony spicule is prevalent in patients with coccydynia, consistent with the findings from previous studies [8, 9]. When a person sits, particularly when leaning partially backward, a bony spicule can irritate the subcutaneous tissue and cause pain [21, 22]. In some authors' description, a pit in the skin overlying the spicule was observed in approximately 60% of cases [21]. This sign was also made in some of our patients (Figure 5), which can be helpful in the diagnosis of coccydynia.

A novel and significant finding was the positive correlation between age and the sacrolumbar angle. An increased sacrolumbar angle alters pelvic tilt and lumbar lordosis, potentially increasing shear forces across the lumbosacral junction and the coccyx itself [8, 23]. Dampc et al. [24] reported the connection between the occurrence of coccygodynia and stenosis of the L5/S1 intervertebral space. Moreover, some patients with coccygodynia may experience pain in the lumbar spinal region [25]. This suggests that coccydynia in older patients may not be an isolated issue but part of a broader postural or degenerative change in the pelvis and spine, warranting a more holistic diagnostic approach.

This study provides a detailed morphological spectrum of the sacrococcyx in patients with coccydynia, offering direct clinical value: significant ventral curvature (Type II/III) or retroversion (t V) of the coccyx, joint subluxation, and bony spicules can serve as important imaging markers for diagnosing idiopathic coccydynia. Moreover, these features contribute to guide personalized treatment decisions: ventral curved types may be more suitable for conservative rehabilitation, while retroverted types, severe dislocations, or those with spicule irritation may require surgical intervention [3, 21, 24]. This study offers a systematic morphological foundation for the imaging diagnosis, personalized treatment, and prognostic evaluation of coccydynia.

The study has several strengths, including a reasonably large patient cohort, comprehensive morphological and morphometric assessments, and a reliable methodology. However, we acknowledge some limitations. Firstly, the study's cross-sectional design and focus solely on symptomatic patients limit our ability to establish causality or distinguish pathological or part of normal anatomical variation. Comparative studies are needed to validate these associations in future research studies. Secondly, all image scans were acquired with patients in a static supine position. Coccygeal movements can occur when transitioning from sitting to standing. For example, increased intrapelvic pressure during sitting may lead to posterior intercoccygeal subluxation [9]. Therefore, dynamic imaging is recommended for patients with suspected coccygeal instability. Thirdly, our data did not include information on stature and body mass index (BMI). Joint subluxation may be more likely in patients with a high BMI, which has been attributed to restricted sagittal pelvic rotation in obese individuals while sitting, resulting in protrusion, retroversion, and excessive pressure on the coccyx's tip [3, 8, 19].

## 5. Conclusion

The study provides a detailed morphological and morphometric characterization of the sacrococcygeal region in patients with coccydynia. The high prevalence of a ventrally curved coccyx, along with features such as bony spicules, lateral deviation, and intercoccygeal subluxation, provides a clear morphological signature for diagnosing idiopathic coccydynia. Furthermore, gender-specific patterns (more curved coccyx in males) and age-related changes (straightening coccyx and increasing sacrolumbar angle) offer valuable insights for individualized risk assessment. Future studies should incorporate comparative cohorts (asymptomatic individuals) and dynamic imaging to elucidate causal relationships between morphology and pain. Despite these limitations, our findings advance the understanding of coccydynia-associated anatomy and may assist the diagnostic refinement and personalized treatment.

## Figures and Tables

**Table 1:** Explanation of sacrococcyx morphology and morphometry parameters.

Parameter	Explanation
*Morphology*	
No. of coccygeal segment	Unfused segment(s) of the coccyx and the fused vertebrae as a single segment.
Types of coccyx (Figure 1)	Type I: slightly curved coccyx pointing downwards
	Type II: more curved coccyx with the apex pointing forwards
	Type III: sharply angulated at intercoccygeal joint
	Type IV: subluxation at the sacrococcygeal or first intercoccygeal joint
	Type V: coccygeal retroversion
Subluxation	Abnormal translation between two adjacent vertebrae
Sacrococcygeal/intercoccygeal joint fusion	Continuity between the bones at the sacrococcygeal/intercoccygeal joint
Coccygeal sacralization	A unilateral or bilateral fusion of the transverse processes of the first coccygeal vertebrae to the inferolateral angle of the sacrum (Figure 2(a))
Coccygeal spicule	A bone projection arising from the terminal coccygeal segment (Figure 2(b))
Lateral deviation of the tip of the coccyx	The angle between the tip of the coccyx and a line passing through the middle of the sacrum greater than 10° (Figure 2(c))

*Morphometry (Figure 3)*	
Coccygeal straight length	The straight-line distance between the midpoint of the upper endplate of first coccygeal vertebra to the coccygeal tip
Coccygeal curved length	The curved-line distance between the midpoint of the upper endplate of first coccygeal vertebra to the coccygeal tip
Sacral or sacrococcygeal straight and curved length	Same as the coccygeal straight and curved length description
Curvature index	Straight length divided by curved length and multiplied by 100
Sacrococcygeal angle	The angle formed by the intersection of a line between the midpoint of the upper endplate of the first sacral vertebra and the first coccygeal vertebra and a line between the latter and the tip of the coccyx
Sacrococcygeal joint angle	The angle between lines intersecting the middle of the last sacral vertebra and the first coccygeal vertebra
Intercoccygeal angle	The angle between lines intersecting the middle of the first and last coccygeal segments in the median plane
Sacrolumbar angle	The angle between the upper border of the first sacral vertebra and the horizontal line

**Table 2:** Morphology parameters of the sacrococcyx in patients with coccydynia and relation to the gender.

Morphology parameters	Total *n* (%)	Sex		*p*
		Male *n* (%)	Female *n* (%)	
Total	242	80	162	
No. of coccygeal segment (s)				0.901
2	20 (8.3)	6 (7.5)	14 (8.6)	
3	108 (44.6)	36 (45)	72 (44.4)	
4	94 (38.8)	30 (37.5)	64 (39.5)	
5	20 (8.3)	8 (10)	12 (7.40)	
Coccyx type				0.976
I	29 (12)	11 (13.8)	18 (11.1)	
II	80 (33.1)	25 (31.3)	55 (34)	
III	68 (28.1)	23 (28.7)	45 (27.8)	
IV	52 (21.5)	17 (21.3)	35 (21.6)	
V	13 (5.4)	4 (5)	9 (5.6)	
Sacrococcygeal subluxation	8 (3.3)	2 (2.5)	6 (3.7)	1.0
Intercoccygeal subluxation				0.697
Total	43 (17.7)	14 (5.8)	29 (11.9)	
Co1–Co2	27 (62.8)	8 (57.1)	19 (65.5)	
Co2–Co3	12 (27.9)	4 (28.6)	8 (27.6)	
Co3–Co4	4 (9.3)	2 (14.3)	2 (6.9)	
Coccygeal sacralization				
Total	75 (31)	26 (10.7)	49 (20.2)	0.721
Bilateral	40 (53.3)	17 (65.4)	23 (46.9)	0.257
Left	19 (25.3)	4 (15.4)	15 (30.6)	
Right	16 (21.3)	5 (19.2)	11 (22.4)	
Sacrococcygeal fusion	37 (15.3)	11 (13.8)	26 (16)	0.64
Intercoccygeal fusion	101 (41.7)	34 (42.5)	67 (41.4)	0.865
Spicules	54 (22.3)	21 (26.3)	33 (20.4)	0.301
Lateral deviation of the coccyx				
Total	47 (19.4)	12 (5)	35 (14.5)	0.22
Left	19 (40.4)	3 (25)	16 (45.7)	0.31
Right	28 (59.6)	9 (75)	19 (54.3)	

**Table 3:** Morphology parameters of the sacrococcyx in patients with coccydynia and relation to the age.

Morphology parameters	Total *n* (%)	Age			*p*
		≤ 20 Y *n* (%)	20–50 Y *n* (%)	> 50 Y *n* (%)	
Total	242	36	168	38	
No. of coccygeal segment(s)					0.129
2	20 (8.3)	3 (8.3)	11 (6.5)	6 (15.8)	
3	108 (44.6)	17 (47.2)	71 (42.3)	20 (52.6)	
4	94 (38.8)	15 (41.7)	71 (42.3)	8 (21.1)	
5	20 (8.3)	1 (2.8)	15 (8.9)	4 (10.5)	
Coccyx type					0.389
I	29 (12)	2 (5.6)	23 (13.7)	4 (10.5)	
II	80 (33.1)	13 (36.1)	50 (29.8)	17 (44.7)	
III	68 (28.1)	12 (33.3)	50 (29.8)	6 (15.8)	
IV	52 (21.5)	6 (16.7)	36 (21.4)	10 (26.3)	
V	13 (5.4)	3 (8.3)	9 (5.4)	1 (2.6)	
Sacrococcygeal subluxation	8 (3.3)	1 (2.8)	6 (3.6)	1 (2.6)	1.0
Intercoccygeal subluxation					
Total	43 (17.7)	6 (2.45)	29 (11.9)	8 (3.3)	0.26
Co1–Co2	27 (62.8)	2 (33.3)	18 (64.3)	7 (77.8)	
Co2–Co3	12 (27.9)	2 (33.3)	8 (28.6)	2 (7.1)	
Co3–Co4	4 (9.3)	2 (33.3)	2 (22.2)	0 (0)	
Coccygeal sacralization					
Total	75 (31)	6 (2.5)	57 (23.6)	12 (5)	0.126
Bilateral	40 (53.3)	4 (66.7)	27 (47.4)	9 (75)	0.201
Left	19 (25.3)	0 (0)	18 (31.6)	1 (8.3)	
Right	16 (21.3)	2 (33.3)	12 (21.1)	2 (16.7)	
Sacrococcygeal fusion	37 (15.3)	2 (5.6)	28 (16.7)	7 (18.4)	0.205
Intercoccygeal fusion	101 (41.7)	13 (36.1)	74 (44)	14 (36.8)	0.546
Spicules	54 (22.3)	5 (13.9)	39 (23.2)	10 (26.3)	0.386
Lateral deviation of the coccyx					
Total	47 (19.4)	3 (1.2)	40 (16.5)	4 (1.7)	0.033^∗^
Left	19 (40.4)	0 (0)	18 (45)	1 (25)	0.327
Right	28 (59.6)	3 (100)	22 (55)	3 (75)	

^∗^ *p* < 0.05.

^∗∗^ *p* < 0.01.

^∗∗∗^ *p* < 0.001.

**Table 4:** Morphometry parameters of the sacrococcyx in patients with coccydynia and relation to the gender.

Morphometry parameters	Total Mean ± SD	Sex		*p*
		Male Mean ± SD	Female Mean ± SD	
Sacral straight length (mm)	135.1 ± 13.77	140.81 ± 11.41	132.28 ± 14	< 0.001^∗^
Sacral curved length (mm)	143.74 ± 13.92	148.48 ± 11.97	141.4 ± 14.26	< 0.001^∗^
Coccygeal straight length (mm)	40.38 ± 7.92	40.31 ± 6.72	40.42 ± 8.46	0.924
Coccygeal curved length (mm)	46.68 ± 9.16	48.53 ± 8.12	45.77 ± 9.52	0.206
Sacrococcygeal straight length (mm)	156.85 ± 19.29	165.19 ± 15.29	152.73 ± 19.77	< 0.001^∗^
Sacrococcygeal curved length (mm)	190.42 ± 18.13	197.02 ± 16.94	187.16 ± 17.86	< 0.001^∗^
Sacral curvature index	94.01 ± 3.63	94.85 ± 2.89	93.59 ± 3.88	0.011^∗^
Coccygeal curvature index	86.98 ± 8.21	83.44 ± 8.05	88.72 ± 7.73	< 0.001^∗^
Sacrococcygeal curvature index	82.37 ± 6.89	83.91 ± 4.61	81.61 ± 7.67	0.014^∗^
Sacrococcygeal angle (°)	113.47 ± 12.76	116.82 ± 12.85	111.81 ± 12.42	0.004^∗^
Sacrococcygeal joint angle (°)	167.96 ± 9.96	170.23 ± 9.02	166.84 ± 10.23	0.012^∗^
Intercoccygeal angle (°)	129.51 ± 18.52	127.84 ± 17.32	130.33 ± 19.08	0.325
Sacrolumbar angle (°)	31.88 ± 12.43	30.52 ± 11.67	32.55 ± 12.77	0.233
Lateral deviation of the coccyx's angle (°)	16.87 ± 6.35	19.56 ± 9.59	15.94 ± 4.62	0.089

*Note:* Data were mean ± standard deviation.

^∗^ *p* < 0.05.

^∗∗^ *p* < 0.01.

^∗∗∗^ *p* < 0.001.

**Table 5:** Morphometry parameters of the sacrococcyx in patients with coccydynia and relation to the age.

Morphometry parameters	Total Mean ± SD	Age			*p*
		≤ 20 Y Mean ± SD	20–50 Y Mean ± SD	> 50Y Mean ± SD	
Sacral straight length (mm)	135.1 ± 13.77	131.61 ± 12.74	136.41 ± 14.09	132.62 ± 12.67	0.079
Sacral curved length (mm)	143.74 ± 13.92	139.5 ± 12.02	144.87 ± 14.37	142.78 ± 13.02	0.099
Coccygeal straight length (mm)	40.37 ± 7.92	35.87 ± 7.25	40.86 ± 7.61	42.48 ± 8.46	< 0.001^∗^
Coccygeal curved length (mm)	46.68 ± 9.16	43.69 ± 9.1	47.09 ± 9.1	47.72 ± 9.13	0.097
Sacrococcygeal straight length (mm)	156.85 ± 19.29	151.27 ± 14.21	157.93 ± 21	157.37 ± 14.42	0.168
Sacrococcygeal curved length (mm)	190.42 ± 18.13	183.19 ± 16.18	191.96 ± 18.83	190.49 ± 15.23	0.031^∗^
Sacral curvature index	94.01 ± 3.6	94.33 ± 3.61	94.19 ± 3.53	92.91 ± 3.79	0.121
Coccygeal curvature index	86.94 ± 8.19	82.84 ± 9.19	87.31 ± 8.01	89.23 ± 6.69	0.002^∗^
Sacrococcygeal curvature index	82.4 ± 6.89	82.68 ± 4.97	82.28 ± 7.58	82.68 ± 5.11	0.92
Sacrococcygeal angle (°)	113.47 ± 12.76	111.45 ± 14.15	113.41 ± 12.62	115.62 ± 11.97	0.373
Sacrococcygeal joint angle (°)	167.96 ± 9.96	165.16 ± 10.79	168.45 ± 10.06	168.45 ± 8.38	0.189
Intercoccygeal angle (°)	129.51 ± 18.52	126.33 ± 20.39	128.89 ± 18.46	135.25 ± 16.04	0.086
Sacrolumbar angle (°)	31.88 ± 12.43	26.62 ± 9.23	32.02 ± 13.4	36.24 ± 8.27	0.003^∗^
Lateral deviation of the coccyx's angle (°)	16.87 ± 6.35	17.7 ± 2.58	16.27 ± 5.18	22.2 ± 14.91	0.202

*Note:* Data were mean ± standard deviation.

^∗^ *p* < 0.05.

^∗∗^ *p* < 0.01.

^∗∗∗^ *p* < 0.001.

## Data Availability

The data that support the findings of this study are available on request from the corresponding author.
